# Prevention of Stomatal Entry as a Strategy for Plant Disease Control against Foliar Pathogenic *Pseudomonas* Species

**DOI:** 10.3390/plants12030590

**Published:** 2023-01-29

**Authors:** Nanami Sakata, Yasuhiro Ishiga

**Affiliations:** Faculty of Life and Environmental Sciences, University of Tsukuba, 1-1-1 Tennodai, Tsukuba 305-8572, Ibaraki, Japan

**Keywords:** epiphytic phase, stomata, virulence factors, plant protection

## Abstract

The genus *Pseudomonas* includes some of the most problematic and studied foliar bacterial pathogens. Generally, in a successful disease cycle there is an initial epiphytic lifestyle on the leaf surface and a subsequent aggressive endophytic stage inside the leaf apoplast. Leaf-associated bacterial pathogens enter intercellular spaces and internal leaf tissues by natural surface opening sites, such as stomata. The stomatal crossing is complex and dynamic, and functional genomic studies have revealed several virulence factors required for plant entry. Currently, treatments with copper-containing compounds, where authorized and admitted, and antibiotics are commonly used against bacterial plant pathogens. However, strains resistant to these chemicals occur in the fields. Therefore, the demand for alternative control strategies has been increasing. This review summarizes efficient strategies to prevent bacterial entry. Virulence factors required for entering the leaf in plant-pathogenic *Pseudomonas* species are also discussed.

## 1. Introduction

Disease outbreaks caused by bacterial pathogens are increasing and threaten food security worldwide. *Pseudomonas syringae* pathovars are categorized as scientifically and economically important plant bacterial pathogens [1]. So far, more than 60 plant-pathogenic *Pseudomonas* pathovars have been isolated that cause a variety of symptoms, including blight, cankers, leaf spots, and galls on different plant species [2]. *P. syringae* and its related bacterial species are divided into 13 phylogroups (PGs) based on multilocus sequence analysis (MLSA) [3]. The 13 PGs are divided into two major categories: the seven late-branching canonical lineages (PGs 1-6 and 10) and the six early branching noncanonical lineages (PGs7-9 and 11-13) [3]. All plant-pathogenic *Pseudomonas* spp. belong to the first category late-branching canonical lineages [3]. A potential pathway of a nonpathogenic *Pseudomonas* strain evolving into a pathogen are summarized in an excellent review [4]. Among these plant-pathogenic *Pseudomonas* spp., each species has characteristics. *P. syringae* pv. *syringae* (*Pss*) B728a (PG4) is a well-adapted epiphyte with a wide host plant range. Such strains can survive and multiply to substantial population levels on healthy host plants, where they are exposed to stressful conditions such as dryness and sunlight [5]. Therefore, *Pss* has been widely used in microbial ecological studies. Conversely, *P. syringae* pv. *tomato* (*Pst*) (PG1a) is a relatively weak epiphyte, but it is a highly aggressive pathogen once inside host tissues [6]. For this reason, *Pst* DC3000 has been used as a suitable plant-pathogenic bacterium for studying bacterial infection mechanisms [6]. Additionally, *Pst* DC3000 infects both tomato and the model plant *Arabidopsis thaliana*, and these advantages have encouraged many researchers to use it in the study of plant–bacterial interactions [6]. Furthermore, the introduction of non-indigenous pathogenic bacteria into several agroecological systems induced devastating agronomical consequences worldwide recently. One of these cases is the kiwifruit canker outbreak, caused by *P. syringae* pv. *actinidiae* (*Psa*) (PG1b) [7]. In particular, *Psa* biovar 3 (*Psa*3) caused devastating damage and spread rapidly to kiwifruit production areas worldwide [8]. Similarly, *P. cannabina* pv. *alisalensis* (*Pcal*) (PG5) is considered an emerging pathogen in Japan since 2009 and is the organism responsible for the severe outbreaks of leaf spot and blight symptoms on cabbage, pak choi, broccoli, Chinese cabbage, red cabbage, and green ball cabbage [9]. 

**Figure 1:**
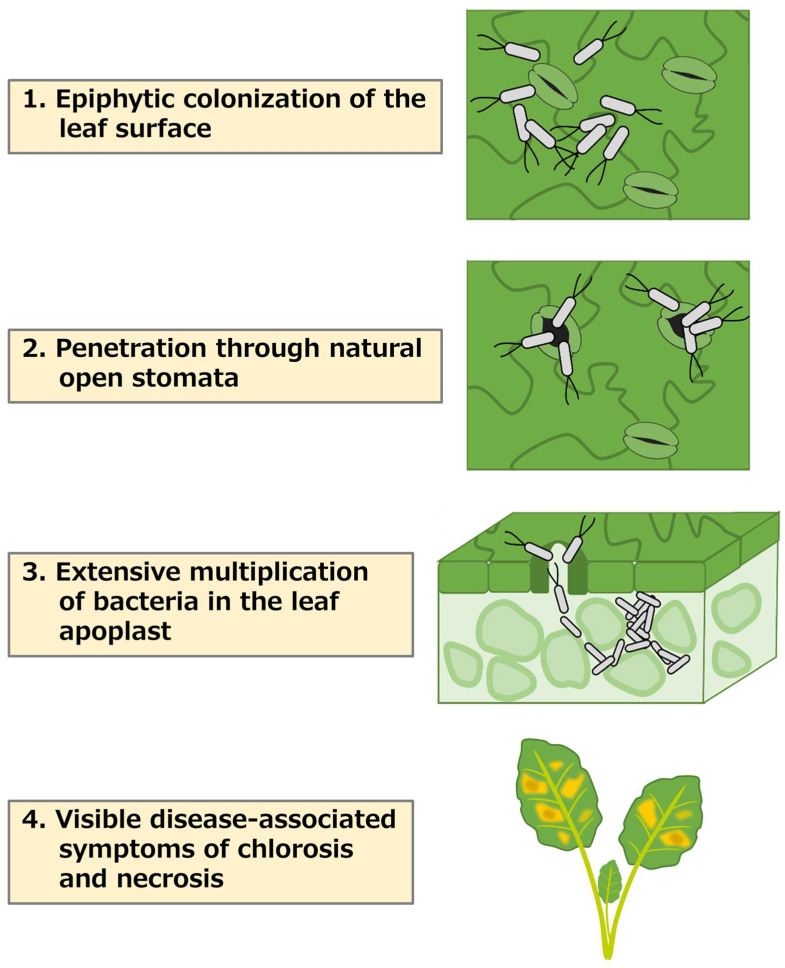
The infection cycle of foliar pathogenic *Pseudomonas* species. On healthy plant leaves, bacteria cells multiply epiphytically (1) and penetrate through open stomata (2). Cross section of a leaf showing bacteria entry, extensive endophytic multiplication, and colonization of the leaf apoplast (3). Visible disease-associated necrosis and chlorosis symptoms (4).

The disease cycle of plant-pathogenic *Pseudomonas* spp. includes: (1) epiphytic colonization of the leaf surface, (2) penetration through natural opening sites such as stomata, (3) extensive multiplication in the leaf apoplast, and (4) visible disease-associated necrosis and/or chlorosis development [6] (Figure 1). Leaf surfaces are relatively suboptimal habitats for bacteria and exhibit strong varying conditions. Water and nutrients are generally lacking and unevenly dispersed on leaf surfaces, and leaves are exposed to high ultraviolet radiation flux and rapid temporal change in temperature, humidity, and water availability [10]. Further, plants have receptors that recognize potential pathogens and activate a wide range of immune responses for self-protection [11]. Perception of a microorganism at the cell surface leads to pathogen-associated molecular patterns (PAMPs)-triggered immunity (PTI). PTI is initiated after the detection of PAMPs by plasma membrane-localized patter recognition receptors (PRRs) [12,13]. Bacteria flagellin (flg22) and EF-Tu (elf18) are recognized by the PRRs FLAGELLIN-SENSING2 (FLS2) and EF-Tu RECEPTOR (EFR), respectively, in *A. thaliana* [12,14]. Moreover, once a pathogen suppresses primary defenses, plants activate a specialized resistance, effector-triggered immunity (ETI) [11]. 

One of the earliest immune responses in PTI is stomatal closure to restrict bacterial entry, so-called stomatal-based defense or stomatal immunity [15,16]. Melotto et al. (2006) [15] showed that stomata can sense PAMPs to close stomata in *A. thaliana*. PRRs on guard cell sense PAMPs and close the stomatal pore [15,16]. Therefore, stomata are not a passive path for pathogen invasion and can prevent pathogen entry into the apoplast. To enter, foliar pathogenic *Pseudomonas* spp. can reopen stomata by using type III secretion effectors (T3Es) or/and phytotoxins. Many recent papers have discussed significant advances toward a mechanistic understanding of stomatal defense and the impact of this discovery on the study of plant–bacterial interactions [17,18,19]. However, disease control strategies targeting infection steps before entry into the plant apoplast have received relatively little attention. Therefore, we here summarize control strategies to prevent stomatal entry of foliar bacterial pathogens. We also summarize the virulence factors involved in the entry of the foliar pathogen *Pseudomonas* spp.

## 2. Virulence Factors Involved in the Entry of Foliar Bacterial Pathogens

Foliar pathogenic *Pseudomonas* spp. enter the plant apoplast through natural opening sites, including stomata, hydathodes, wounds, and lenticels. One of the earliest immune responses in PTI is a stomatal-based defense to restrict bacterial entry through stomata [15]. Thus, stomata are not a passive path for pathogen invasion. For successful entry, foliar pathogenic *Pseudomonas* spp. need to move toward natural opening sites such as stomata and overcome plant early defense PTI. Thus, we here summarized bacterial virulence factors required for stomatal entry.

### 2.1. Motility

**Figure 2:**
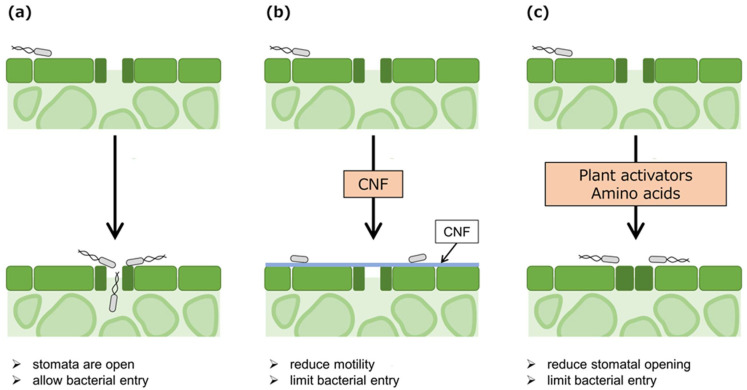
A diagram describing successful and failed entry into plants. (**a**) Bacteria overcome stomatal-based defense by using phytotoxins and type 3 effectors, resulting in successful entry. (**b**) Covering the leaf surface with cellulose nanofibers (CNFs) leads to motility reduction, limiting bacterial entry. (**c**) Plant activators (e.g., acibenzolar-*S*-methyl and probenazole) and amino acids (e.g., cysteine, glutamic acid, and lysine) lead to a reduction in stomatal aperture, limiting bacterial entry.

Flagella and pili are required for bacterial motility. The importance of motility in the plant disease cycle and thus in virulence in *Pseudomonas* spp. was reported since the 1970s [20]. For successful entry of foliar pathogenic *Pseudomonas* spp., bacteria move forward to natural opening sites by flagella and type IV pili (T4P) (Figure 2a). Motility loss due to flagella-related gene mutation remarkably decreased virulence in plant-pathogenic *P. savastanoi* pv. *phaseolicola* (*Psp*), *Pss*, and *P. savastanoi* pv. *glycinea* (*Psg*) [20,21,22]. Mutants in a flagellin-encoded gene, Δ*fliC*, lost motility and reduced disease symptom development and bacterial multiplication in *Pst* DC3000, *P. amygdali* pv. *tabaci* (*Pta*) 6605, and *Pcal* KB211 after spray inoculation [23,24,25,26]. However, after syringe inoculation, a Δ*fliC* in *Pta* 6605 exhibited reduced virulence [23], but Δ*fliC* in *Pst* DC3000 grew similarly to wild-type [27]. Disease symptoms and the bacterial population size of Δ*fliC* in *Pcal* KB211 were also not significantly different after syringe inoculation (Sakata et al. unpublished data). Studies on Δ*fliC* in *Pta* 6605 suggested that motility loss resulted in the dramatic reduction in *N*-acyl homoserine lactones (AHLs), pyoverdine, major first siderophores, and biofilm formation [28,29]. Flagellin glycosylation in *Pta* 6605 is required for the stability of flagella filaments, flagellin polymerization, proper motility, and virulence promotion [30]. *Pta* 6605 Δ*fgt1* and Δ*fgt2* (mutants defective in the *flagellin glycosyltransferase* genes *1* and *2*, respectively) showed reduced virulence after spray inoculation, but not after syringe inoculation [31]. These results highly supported that flagellar motility is important in the epiphytic phase and for bacterial entry. Although motility loss caused reduced virulence in several plant-pathogenic *Pseudomonas* spp., *Pst* DC3000 has a few flagella and decreased flagellar motility compared with *Pta* 6605 [30]. Therefore, the differential contribution of flagellar motility to virulence among several pathovars should be considered.

Based on the *P. aeruginosa* sequence, flg22, a 22-amino acid epitope of FliC, is sufficient to induce PTI in *A. thaliana* [32,33]. Flg22 is extremely well-conserved in the *Pseudomonas* genus including animal and plant pathogens [32]. Despite this extreme conservation, some pathogens have polymorphic flg22 epitopes that avoid PTI. Indeed, the flg22 allele of *Pcal* ES4326 is inactive as a PAMP but acts as an antagonist for flg22 [34]. Parys et al. (2021) [35] investigated how single amino acid changes in the immunogenic flg22 motif affect bacterial motility and the interaction with the *A. thaliana* immune receptor FLS2. Mutations in the first 17 amino acids of the flg22 peptide, representing the “address” segment important for the interaction with FLS2, had the strongest impact on motility function [35]. Mutations in the last five amino acids, representing the “message” segment important for BAK1 (BRASSINOSTEROID INSENSITIVE 1-associated receptor kinase 1) docking, did not affect motility [35]. The impact of the flg22 epitopes concerning the interaction of PTI avoidance and motility needs further investigation.

Twitching motility is generally thought of as T4P movement. Although T4P provides an advantage to bacteria in surface motility (called twitching), surface adherence, colonization, and biofilm formation in animal pathogenic bacteria [36], investigation of T4P as a virulence factor has been limited so far in plant-pathogenic bacteria. Mutants in T4P encoded genes (including a *pilA* mutant) were not impaired in swimming motility in a liquid medium, but they showed remarkably reduced swimming and swarming motility in a semisolid medium, indicating that T4P are required for surface motility in *Pta* 6605 [37]. However, a *Pst* DC3000 *pilA* mutant exhibited reduced swimming motility and increased swarming motility in a semisolid medium and reduced bacterial population *in planta* [38]. T4P might function differentially between these two pathovars, T4P in *Pst* DC3000 contribute to UV tolerance and are important in epiphytic survival [38], and mutants in T4P in *Pta* 6605 exhibited full virulence after spray inoculation [37]. Moreover, the type IV secretion system was identified from several screenings as a virulence factor in *Pcal* KB211 [39] and *Psa*3 [40]; therefore, T4P contribute to plant leaf interactions in several *Pseudomonas* spp. during infection.

### 2.2. Taxis

Chemotaxis allows bacteria to move toward or away from environmental cues, facilitating bacterial entry through stomata and wounds [41]. Chemotaxis is essential for establishing beneficial plant-bacteria interactions [42], but also has important roles for pathogenic bacteria. Chemotaxis is very important for plant invasion, as *Pst* DC3000 exhibits chemotaxis toward open but not closed stomata [15,41], and chemotaxis genes, including several chemoreceptors, were upregulated in epiphytic cells and repressed in apoplastic cells [43].

Despite the importance of motility and chemotaxis in *Pst* DC3000 colonization and entry, only two of its 49 chemoreceptors were characterized. The amino acid receptor PscA bound and mediated chemoattraction to D-aspartic acid [Asp], L-Asp, and L-glutamic acid [Glu], and was required for full virulence in tomato [44]. PscC binds gamma amino butyric acid [GABA] and L-proline [Pro], two abundant components of the tomato apoplast, and was also required for full virulence [45]. A *pscC* mutant showed reduced entry, resulting in reduced populations after spray inoculation compared with the wild-type, but no significant differences were observed when plants were infiltrated [45]. Therapeutic strategies for interfering with chemotactic signaling pathways may block bacterial pathogen entry and prevent disease [41].

Bacterial aerotaxis is a rapid response towards or away from oxygen [46]. Aerotaxis was also required for early colonization in host plants and biofilm formation in the foliar bacterium *Pta* 6605 [47]. Further research on chemotaxis and aerotaxis elucidate how bacteria respond to other plant signals as cues to enter the plant apoplast and cause disease.

### 2.3. Phytotoxins

Phytotoxins are produced during infection and generally injure plant cells and affect disease symptom development. In plant-pathogenic *Pseudomonas* spp., coronatine (COR) and syringolin A are important in the epiphytic phase.

COR is composed of the polyketide coronafacic acid and coronamic acid [48,49,50]. COR structurally mimics jasmonic acid-isoleucine (JA-Ile), an active form of jasmonic acid (JA). COR binds the COI1-JAZ (CORONATINE INSENSITIVE1-JASMOTATE ZIM-DOMAIN) coreceptor, then activates transcriptional factors MYC2/NAC, leading to suppression of PAMPs-induced abscisic acid (ABA)-mediated stomatal closure [17]. Moreover, COR triggers stomata to reopen via endoplasmic reticulum-mediated function independent of COI1-JAZ [51], facilitating bacterial entry. COR also has multiple roles during infection, including promoting bacterial multiplication, persistence *in planta*, and disease symptom induction [15,52,53,54,55,56,57,58].

Syringolin A, produced by *Pss*, is a product of a mixed nonribosomal peptide and polyketide synthetase [59]. A *Pss* syringolin A-negative strain showed reduced virulence on common bean (*Phaseolus vulgaris*) compared with the wild-type, indicating that syringolin A is an important virulence factor [60]. Syringolin A-producing bacteria can open stomata and thus counteract stomatal-based defense in bean and *A. thaliana* [61].

### 2.4. Type Three Secretion System

In a successful case of *Pcal* entry, *Pcal* reopens stomata by secreting COR and type three effectors (T3Es) [58] (Figure 2a). The type three secretion system (T3SS) in plant-pathogenic *Pseudomonas* is encoded by the *hypersensitive response and pathogenicity* (*hrp*) genes, which are induced by the sigma factor HrpL. HrpR and HrpS form a hetero-hexameric transcriptional factor and activate *hrpL* expression [62,63]. Revealing the individual effector function was difficult due to the diverse and internally redundant effector repertoires. Therefore, Cunnac et al. (2011) [64] constructed a functionally effectorless derivative of *Pst* DC3000, designated DC300028E, and identified the minimal function repertoire of T3Es that are required for disease symptom formation and bacterial multiplication [64]. Further, HopX1, HopBB1, and HopZ1 are functionally redundant with COR [65,66,67]. Chakravarthy et al. (2018) [68] demonstrated that functionally effectorless *Pst* DC3000 derivatives that were restored for COR production and two key effectors, HopM1 and AvrPtoB, produce disease symptoms [68]. Further, AvrPto, AvrPtoB, HopB1, HopF2, AvrRpt2, and AvrRpt4 suppress PTI, including stomatal-based defense [69,70,71,72,73,74,75,76,77,78]. *Psa*3 *hopR1* mutants failed to reopen stomata on kiwifruit leaves, and exhibited significantly reduced virulence, suggesting that the T3E HopR1 facilitates stomatal entry [40].

## 3. Strategies to Prevent the Entry of Foliar Bacterial Pathogens

Plant bacterial diseases are severe problematic issues, and few resources are sufficient to mitigate crop loss. Currently, chemical treatments such as copper-containing fungicides and antibiotics that reduce bacterial numbers on plants are common strategies used against bacterial pathogens. Antibiotics (such as streptomycin, oxytetracycline, gentamycin, and oxolinic acid) are used for plant protection [79]. Unfortunately, streptomycin resistance in plant pathogens was detected within five to ten years of the antibiotic commercialization [79,80]. Since the initial use of copper-containing fungicides to prevent downy mildew since the end of the 19th century, many copper-based antimicrobial compounds have been applied for crop protection [81]. The number of resistance strain reports has markedly increased since the 1980s [82,83]. In Japan, *Pcal* strains resistant to streptomycin and copper-containing fungicide have been isolated [84]. Therefore, the demand for efficient and sustainable alternative bacterial disease control strategies has been increasing.

**Figure 3:**
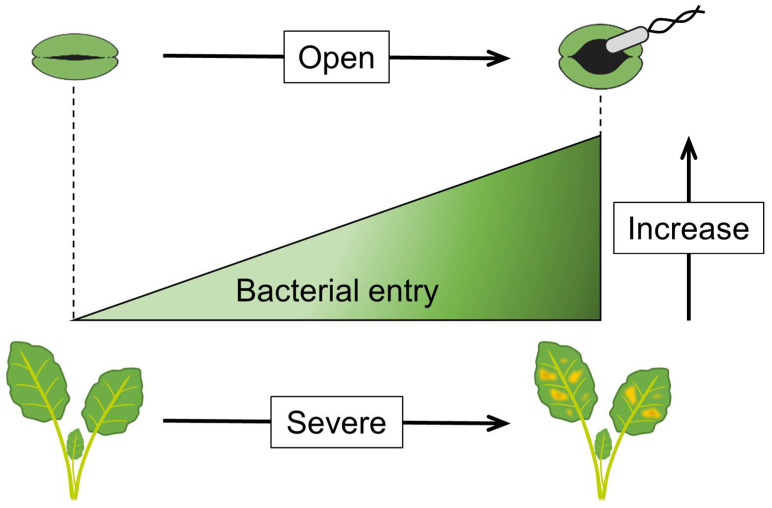
The correlations among stomatal opening width, bacterial entry, and disease symptoms. As stomata is open, bacterial entry increases. As the initial bacterial entry increases, severe disease symptoms occur.

If initial bacterial entry was related to visible lesion formation, we would expect disease severity to be as well. Reducing stomatal width can limit bacterial entry into plants, leading to reduced disease symptoms [85] (Figure 3). Indeed, the strategies outlined below to prevent the entry of foliar bacterial pathogens are effective disease control strategies.

### 3.1. Cellulose Nanofibers

Cellulose nanofibers (CNFs) can be produced from cellulose, which is one of the most abundant and renewable biomass sources in nature. CNF derived from the aqueous counter hydrolysis (ACC) method has amphipathic properties, which converts the properties of treated surfaces from hydrophobic to hydrophilic, and vice versa [86]. Covering cabbage leaves with CNF suppressed bacterial blight caused by *Pcal* KB211 [26]. Notably, expression of the bacterial flagellin-encoded gene, *fliC*, in *Pcal* KB211 was also downregulated on leaf surfaces covered with CNF which decreases motility, significantly reducing bacterial entry [26] (Figure 2b). Moreover, nanofibers such as chitin nanofibers induce plant resistance by activating defense-related genes [87]. However, CNF did not induce plant-defense genes, indicating that CNF does not have elicitor activity [26]. Altering leaf surface properties via CNF can be a novel and efficient strategy for preventing bacterial entry, and thus controlling bacterial diseases.

### 3.2. Plant Defense Activators

Systemic acquired resistance (SAR) processes can be divided into three steps: local immune activation, information relay from local to systemic tissues by mobile signals, and defense activation and priming in systemic tissues [88,89,90]. The establishment of salicylic acid (SA) as the endogenous signal for SAR prompted the development of SA analogs. Plant defense activators are attractive compared with conventional pesticides, and the capacity of pathogens to select for resistance to these chemicals due to their broad-spectrum protective effects is low [91].

Acibenzolar-*S*-methyl (ASM), which is a synthetic analog of SA, showed a protective effect against various pathogens, including fungi, bacteria, and viruses [92,93,94,95]. ASM induces resistance systemically by acting downstream of SA without SA accumulation [96]. In Japan, ASM has been used since 2020 for bacterial blight, caused by *Pcal*, on cabbage and Chinese cabbage. Soil drenched with ASM suppressed *Pcal* KB211 disease development on leaves within 2 h after ASM treatment [97]. Since ASM showed a rapid protective effect, it seemed to affect the early infection process. Further work revealed that ASM activated stomatal-based defense against *Pcal* KB211 [97], reducing stomatal width and limiting bacterial entry into the plant apoplast [97] (Figure 2c). The ASM-triggered stomatal closure was observed both in dicotyledoneae and monocotyledoneae plants [97,98,99,100]. This finding reveals a novel mechanism of ASM protection against bacterial pathogens.

Probenazole (PBZ) also induces the SA signaling pathway the same as ASM, but PBZ acts in the step before SA biosynthesis [101,102]. PBZ has been widely used to protect rice from the rice blast fungus *Pyricularia oryzae* in Asia, and from the bacterial blight pathogen *X. oryzae* pv. *oryzae* [103]. PBZ soil drench also induced stomatal-based defense against *Pcal* KB211 in cabbage [100]. Moreover, PBZ also induced resistance systemically by stimulating the SA/NPR1 (NON-EXPRESSOR OF PR GENES 1)-mediated defense signaling pathway upstream of SA biosynthesis in the dicotyledoneae plant *A. thaliana* [102]. Therefore, PBZ is also an effective plant defense activator to control bacterial disease in various crops.

Noutoshi et al. (2012) [104] conducted a high-throughput quantitative screen to identify plant immune-priming compounds that potentiate but not directly induce immune responses. They screened for compounds that specifically potentiate pathogen-activated cell death in *A. thaliana* cell suspension cultures, and identified five novel compounds that enhanced disease resistance in plants [104]. Since one of the protection mechanisms of ASM is to activate stomatal-based defense, “modulating stomatal movements” is a novel target for developing control strategies against pathogens. Indeed, chemical screening for compounds that regulate stomatal movements was conducted and identified compounds that triggered stomatal closure [105]. Screening for compounds that enhance disease resistance represent a novel way of controlling plant-pathogenic diseases.

### 3.3. Amino Acids

While bacteria often require amino acid receptors for full virulence, amino acids have been used as water-soluble fertilizers to promote plant growth and improve plant quality [106,107,108,109]. Additionally, amino acid application induces plant resistance. For instance, exogenous treatment with glutamic acid [Glu] enhanced plant resistance against the fungal pathogen *P. oryzae* in rice [110], *Alternaria alternata* in tomato [111], *Colletotrichum higginsianum*, and the foliar pathogen *Pst* in *A. thaliana* [112] and *Pcal* in cabbage [85]. One of the protection mechanisms of amino acids against *Pcal* KB211 is reducing stomatal aperture and limiting bacterial entry [85] (Figure 2c). Several amino acids (e.g., Cysteine [Cys], Glu, and Lysine [Lys]) reduced stomatal aperture and limited bacterial entry [85] (Figure 2c). Cys triggered stomatal closure by inducing abscisic acid (ABA) biosynthesis [113]. Moreover, amino acids, which showed a protective effect against *Pcal* KB211, suppressed disease symptoms and bacterial populations after spray inoculation but not syringe inoculation, indicating that they mediated a protective effect in the epiphytic phase before the pathogen entered plants [85]. These results indicate that amino acids also confer a protective effect by preventing bacterial entry to control disease. Natural compounds, including amino acids, can be important in sustainable agriculture.

Growth-defense tradeoffs are thought to occur in plants due to resource restrictions [114]; what about growth-virulence tradeoffs in plant pathogens? Sakata et al. (2021) [115] demonstrated that a *trpA* mutant (disrupted in tryptophan synthase alpha chain) exhibited significantly reduced virulence. TrpA was necessary for bacterial growth both on leaf surfaces and in the apoplast. Moreover, the *trpA* mutant showed reduced expression of COR and T3SS-related genes [115]. This study indicates that a tryptophan deficiency in bacteria leads to a reduction in virulence. By controlling nutrients such as amino acids on leaf surfaces, pathogenic bacteria might become undernourished, leading to virulence reduction.

## 4. Conclusions and Prospects

During successful infection in *Pseudomonas* spp., stomatal entry is a critical step that determines infectivity. Several control strategies prevent bacterial entry into plants, such as cellulose nanofiber, plant activators, and amino acids, and are efficient ways for controlling bacterial diseases. Most foliar bacterial pathogens target stomata as the main entry site. Therefore, defending against pathogen infection before stomatal entry is a powerful strategy to suppress plant diseases. To investigate whether these strategies showed protective effect against other bacterial pathogens should be tested. Moreover, a field trial is needed to be put these strategies into practical use. Continuous use with antibiotics and copper-containing fungicides promotes appearance of resistance strains and has a negative impact on the environment. Thus, alternative strategies introduced here and other natural materials can be solutions in realizing sustainable and eco-friendly agriculture.

## Data Availability

Not applicable.
